# Retrospective 25-year follow-up of treatment outcomes in Angle Class III patients: Success versus failure

**DOI:** 10.1007/s00056-016-0075-8

**Published:** 2017-02-15

**Authors:** Brigitte Wendl, A. Kamenica, H. Droschl, N. Jakse, F. Weiland, T. Wendl, M. Wendl

**Affiliations:** 1 Clinical Department of Oral Surgery and Orthodontics, Medical University Graz, Billrothgasse 4, 8036 Graz, Austria; 2 Deutschlandsberg, Austria; 3 University Dental School Vienna, Vienna, Austria; 4 Institute of Software Development and Biomedical Engineering, Technical University Graz, Graz, Austria

**Keywords:** Class III therapy, Prognostic parameters, Treatment success, Chincup, Klasse-III-Therapie, Prognostische Parameter, Therapieerfolg, Kopf-Kinn-Kappe

## Abstract

**Objectives:**

Despite recommendations for early treatment of hereditary Angle Class III syndrome, late pubertal growth may cause a relapse requiring surgical intervention. This study was performed to identify predictors of successful Class III treatment.

**Materials and methods:**

Thirty-eight Class III patients treated with a chincup were retrospectively analyzed. Data were collected from the data archive, cephalograms, and casts, including pretreatment (*T*0) and posttreatment (*T*1) data, as well as long-term follow-up data collected approximately 25 years after treatment (*T*2). Each patient was assigned to a success or a failure group. Data were analyzed based on time (*T*0, *T*1, *T*2), deviations from normal (Class I), and prognathism types (true mandibular prognathism, maxillary retrognathism, combined pro- and retrognathism).

**Results:**

Compared to Class I normal values, the data obtained in both groups yielded 11 significant parameters. The success group showed values closer to normal at all times (*T*0, *T*1, *T*2) and vertical parameters decreased from T0 to T2. The failure group showed higher values for vertical and horizontal mandibular growth, as well as dentally more protrusion of the lower anterior teeth and more negative overjet at all times. In adittion, total gonial and upper gonial angle were higher at *T*0 and *T*1. A prognostic score—yet to be evaluated in clinical practice—was developed from the results. The failure group showed greater amounts of horizontal development during the years between *T*1 and *T*2. Treatment of true mandibular prognathism achieved better outcomes in female patients. Cases of maxillary retrognathism were treated very successfully without gender difference. Failure was clearly more prevalent, again without gender difference, among the patients with combined mandibular prognathism and maxillary retrognathism. Crossbite situations were observed in 44% of cases at *T*0. Even though this finding had been resolved by *T*1, it relapsed in 16% of the cases by *T*2.

**Conclusion:**

The failure rate increased in cases of combined mandibular prognathism and maxillary retrognathism. Precisely in these combined Class III situations, it should be useful to apply the diagnostic and prognostic parameters identified in the present study and to provide the patients with specific information about the increased risk of failure.

## Zusammenfassung

**Einleitung:**

Das Angle Klasse-III-Syndrom ist eine vererbbare Dysgnathie, bei der eine Frühtherapie empfohlen wird. Dennoch kann es durch spätpubertäres Wachstum zu einem Rezidiv kommen, und ein kieferchirurgischer Eingriff kann notwendig werden.

**Ziel:**

Ermittlung prognostischer Parameter für eine erfolgreiche Klasse-III-Behandlung im Rahmen der Ausgangsdiagnostik.

**Material und Methode:**

Retrospektiv analysiert wurden die Daten von 38 Patienten mit therapeutisch abgeschlossenem Klasse-III-Syndrom. Untersucht wurden vor der Behandlung ausgefüllte Anamnesebögen, Fotos, Fernröntgenaufnahmen und Modelle. Der Behandlungserfolg wurde durch Datenerhebung bei Therapieende und einer Abschlussanalyse etwa 25 Jahre nach Therapie ermittelt. Es erfolgte eine Einteilung in eine Erfolgs- und in eine Misserfolgsgruppe. Die Datenanalyse basierte auf unterschiedlichen Zeitintervallen, dem Alter, Normwertabweichungen und der Form der Progenie.

**Ergebnisse:**

Verglichen mit Klasse I-Patienten zeigten beide Gruppen in 11 Parametern signifikante Unterschiede. Die Werte in der Erfolgsgruppe waren bei Therapieanfang, bei Therapieende und bei der Nachuntersuchung dem Normwert näher. Gleichzeitig nahmen die vertikalen Parameter von T_0_ nach T_2_ ab. In der Misserfolgsgruppe zeigte der Unterkiefer stärkeres Wachstum in der Länge und in der Höhe. Der Gesamtgonionwinkel und der obere Gonionwinkel waren vor der Therapie in der Misserfolgsgruppe größer. Dental wies die Misserfolgsgruppe eine protrudiertere Unterkiefer-Frontzahnstellung und einen stärkeren negativen Overjet auf. Aus den Ergebnissen wurde ein Prognosescore entwickelt, der in seiner klinischen Anwendung zukünftig weiter evaluiert werden muss. Im Intervall zwischen Therapieende und Nachuntersuchung war in der Misserfolgsgruppe eine stärkere horizontale Entwicklung zu erkennen. Bei der Einteilung nach Progenieform zeigte sich, dass bei der "echten Progenie" (mandibulären Prognathie) Mädchen mit mehr Erfolg therapiert wurden. Die maxilläre Retrognathie konnte sehr erfolgreich behandelt werden, zwischen den Geschlechtern ergab sich hier kein signifikanter Unterschied. Bei der Behandlung von Patienten mit einer Kombination aus mandibulärer Prognathie und maxillärer Retrognathie zeigten sich unabhängig von Geschlecht deutlich mehr Misserfolge. Vierundvierzig Prozent der untersuchten Patienten hatten bei Therapiebeginn einen Kreuzbiss, der bei Therapieende zwar korrigiert war, zum Zeitpunkt der Nachkontrolle jedoch bei 16% der Fälle rezidiviert war.

**Schlussfolgerung:**

Die Frühbehandlung von Patienten mit Kombinationsformen der Progenie hatte eine geringere Erfolgsquote. Hier sollten die in dieser Studie ermittelten diagnostischen und prognostischen Parameter verwendet werden und eine entsprechende Patientenaufklärung erfolgen.

## Introduction

Angle Class III malocclusion is one of the greatest challenges in orthodontics. Its documented global prevalence varies widely, including 4–13% of the Japanese population as reported by Litton et al. [21], 6% of Swedes versus 0.8% of white and 0.6–1.2% of black Americans as referred to by Nakasima et al. [25, 26], or 1.8% of Austrians as reported by Droschl [7]. Angle Class III malocclusion is a hereditary syndrome capable of assuming different severities and of skipping generations. An epigenetic trigger has also been implicated in its causation [7, 12, 14, 32]. One of the findings from numerous studies available on the subject is that greater skeletal and dental changes toward Class I can be achieved when orthodontic treatment is performed early rather than late [5, 13, 20].

An early—or timely—diagnosis already in the primary dentition stage is essential to prevent the genetic disposition from becoming manifest. Early treatment of true mandibular prognathism is about recognizing existing anatomical limitations and avoiding progression. Yet once a situation turns out to be treatment-resistant, the early strategy should be abandoned for a combined orthodontic and orthognathic surgical approach to be performed after completion of growth [17]. Still, many Class III patients need retreatment after early orthodontic treatment due to discrepant maxillary and mandibular growth during the pubertal growth spurt [23]. Ngan et al. [27] showed that, after a 4-year observation period following successful completion of facemask treatment, 25% of patients again presented an inverted overbite. Sugawara et al. [33] similarly reported that many outcomes of chincup treatment were unstable during pubertal growth. Other authors [15, 18] have suggested that growth changes, and hence outcomes, vary from patient to patient.

The question arises in what situations treatment should be started in early childhood as opposed to adopting a wait-and-see strategy and performing orthognathic surgery at a later time. Extensive research has gone into modifiers of relapse and predictors of success to allow for better forecasts of treatment outcomes and long-term stability [1–3, 8, 9, 11, 19, 24, 27, 29, 31, 35–37]. However, the parameters that were used across these studies varied widely. Some of them have been analyzed in a review by Fudalej et al. [10], who evaluated 14 studies for predictors and identified 38 variables. Most authors used combinations of three or four parameters for prognosis, gonial angle and Wits appraisal being the most common, followed by mandibular length and the SNA, SNB, and ANB angles. Johnston [16] devised a simple “forecast grid” to predict growth based on mean-value increases of some cephalometric parameters.

Schulhof et al. [30] evaluated the parameters of molar relationship, cranial deflection, porion, and ramus location on cephalometric tracings to predict normal or abnormal growth. A longitudinal study by Franchi et al. [9] on patients treated with a chincup found that crucial parameters for successful outcomes were inclination of condylar axis to basocranial plane, inclination of the maxillary plane to the mandibular line, and transverse mandibular width. Prognostic variables reported in a Japanese study included gonial angle, position of mandible relative to the cranial base, N-A-Pg, and angle from ramus line to SN line [35]. A 1995 study by Battagel and Orton [3] showed four significant variables to forecast relapse after non-extraction treatment of Class III malocclusion, including anterior maxilla to maxillary plane, labrale inferius to sella vertical line, labrale superius to soft-tissue nasion, and number of anterior teeth in crossbite.

We performed this retrospective study to identify relevant cephalometric, dental, and anamnestic parameters by comparing a success and a failure treatment group of Class III malocclusion patients.

## Materials and methods

Pre- and posttreatment anamnestic records, cephalograms, and casts were analyzed for this study, which comprised 38 female and male Class III patients who had received chincup therapy and were followed up after approximately 25 years. Crossbites had been corrected with a cemented acrylic expansion device. We only included patients for whom complete pretreatment (*T*0), posttreatment (*T*1), and follow-up (*T*2) documentation was available and who had presented skeletal and dental Class III syndrome at *T*0, at this point they were 5–10 years old. Cleft disease or any other syndromes led to exclusion, and we did not include patients who had undergone orthognathic surgery.

**Fig. 1:**
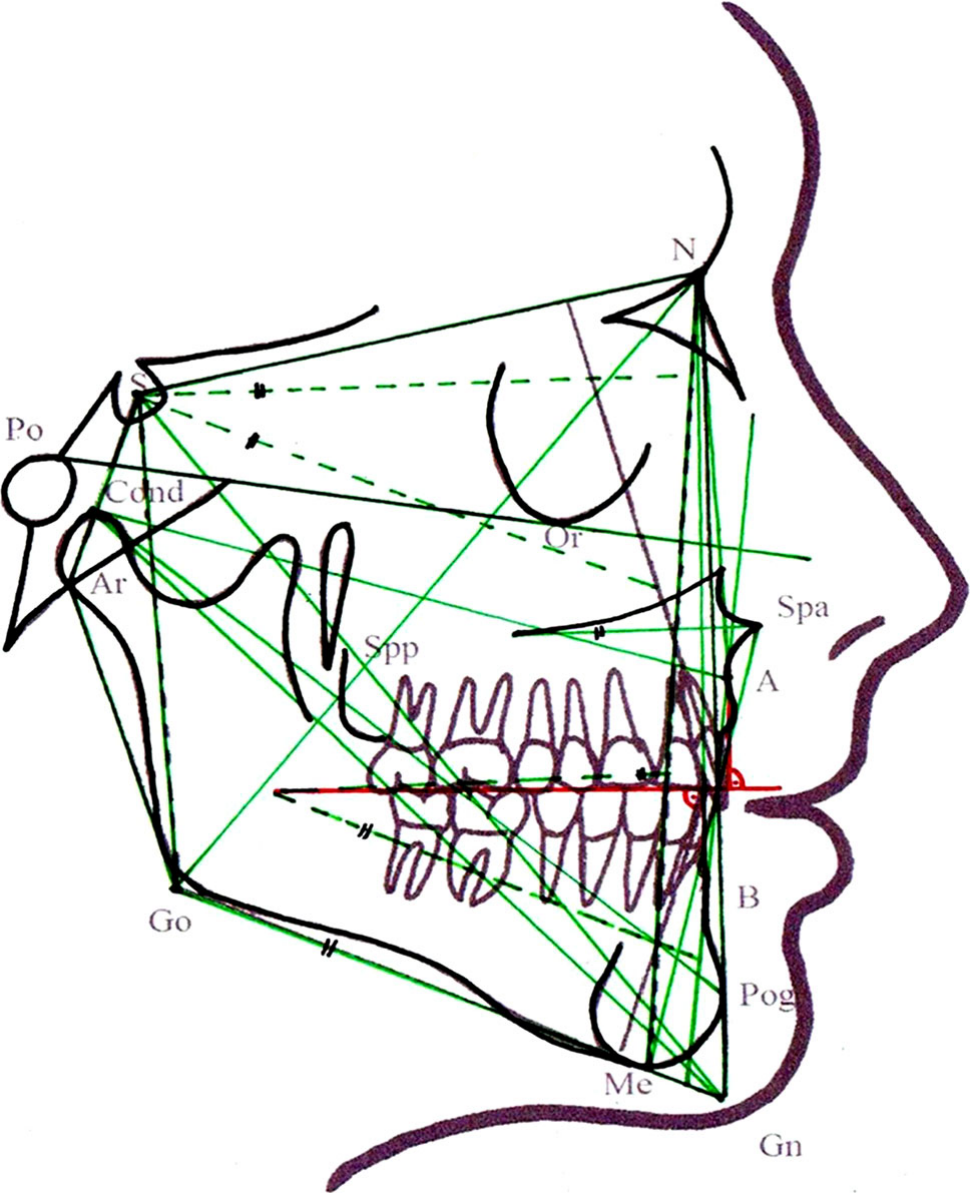
Analysis of the lateral cephalogram with 37 evaluated parameters (linear and angular measurements) **Abb. 1** Fernröntgenseitenbildanalyse mit den 37 ausgewerteten Parametern (lineare und Winkelmessungen)

Patients were assigned to a success or failure group based on the results of the *T*1 and *T*2 examinations. Figure 1 illustrates the 37 linear and angular cephalometric parameters that were measured on each patient’s *T*0, *T*1, and *T*2 cephalograms for analysis and comparison. Overbite, overjet, and transverse upper and lower jaw width were measured on the casts. Criteria for assignment to the success group were positive overjet and overbite (≥1 mm) and no transverse crossbite. The resultant success group included 25 (12 female and 13 male) and the failure group 13 (2 female and 11 male) patients. Control data of normal Class I patients were only needed to statistically calculate deviations from normal, considering that the study was mainly designed to compare a success and a failure group (based on different examination times, deviations from normal, and prognathism types). We therefore relied on normal values from the literature, which were representative of our patient sample—also reflecting the changes with age.

To evaluate associations between treatment success and specific Class III patterns, we distinguished between true mandibular prognathism, maxillary retrognathism, and combined cases of mandibular prognathism and maxillary retrognathism based on normal values from the literature. The intraclass correlation coefficient (ICC) for errors of measurement, tracing and assignment committed by two experienced examiners was 0.989, thus, indicating high agreement. SPSS (Version 22″, 2013) software was used for descriptive and explorative data analysis. Differences were considered significant at *p* ≤ 0.05. A *t* test for independent samples and one-way ANOVA were applied to compare mean values, and the ICC was calculated for each parameter to judge the tracing precision of the examiners.

## Results

Table 1 lists the descriptive statistical results for the 37 cephalometric parameters, classified by success versus failure group and examination times. The failure group comprised 13 patients (4 failures at *T*1 and 9 at *T*2) and, compared to the success group, showed higher values for 1) mandibular growth, with pronounced changes in mandibular length (Con-Gn, Cond-Pg, Go-Me) and ramus height; 2) for SNB angle at *T*1 and *T*2, with no significant difference at *T*0; 3) for gonial and upper gonial angle at *T*0 and *T*1, although decreasing over the course of treatment; 4) for maxillomandibular difference; 5) anterior posterior dysplasia indicator (APDI) at *T*0, *T*1, and *T*2; 6) for cranial base angle, although this difference was not statistically significant; 7) for upper incisor inclination showing greater indications of camouflage (i.e., protrusion of the maxillary anterior segment) at *T*1 and *T*2; and 8) the lower anterior teeth were more protruded.

**Tab. 1:** Descriptive statistical pretreatment (*T0*), posttreatment (*T1*) and 25-years follow-up (*T2*) data obtained in the success versus failure group for the 37 cephalometric parameters measured **Tab. 1** Deskriptive Statistik der 37 Fernröntgenwerte in der Erfolgs- und in der Misserfolgsgruppe zu den Zeitpunkten *T*0 (vor Therapie), *T*1 (nach Therapie) und *T*2 (25 Jahre nach Therapie)

Wits (mm)	Pretreatment values (*T*0)				Posttreatment values (*T*1)				25-year follow-up (*T*2)			
	Failure		Success		Failure		Success		Failure		Success	
	Mean	SD	Mean	SD	Mean	SD	Mean	SD	Mean	SD	Mean	SD
	−4	3.54	−2.83	2.7	−2.43	2.51	−1.83	2.71	−2.57	3.16	−2.89	3.45
GH (%)	61.29	1.89	62.5	3.33	65.71	4.07	64.17	6.6	69.57	1.99	67.61	5.39
SNA	76.71	3.77	78.17	3.26	79	1.53	78.44	4.27	81	3.32	78.67	4.65
SNB (°)	78.5	2.5	77.29	2.87	79.29	3.35	78.22	4.26	83	4.62	79.83	3.87
ANB (°)	2.43	1.72	1.56	1.34	3	1.83	1.78	1.56	3.43	2.76	1.83	2.18
Go (°)	133.14	4.49	130	5.42	128.86	5.73	125	6.89	123.43	5.97	123.06	6.05
Börk’s sum (°)	395.71	3.25	394.22	4.85	378.13	40.55	388.11	24.3	388.43	6.5	387.61	18.24
Gn/SN (°)	67.86	4.53	65.56	3.05	66	2.71	65.61	5.15	63.29	4.03	65.78	3.28
Spp-Spa (mm)	48.14	2.79	47.67	4.27	50	3.83	51.22	3.77	54.72	2.23	56.57	4.75
Cond-A (mm)	78.24	4.75	80.29	4.97	83.65	5.26	86.43	5.31	89.11	4.41	92.14	4.69
Cond-Gn (mm)	112.29	10.09	108.24	6.63	124.57	12.11	118	8.97	135.14	13.83	126.56	9.65
MM differential (mm)	32	9.5	29.29	6.25	38.14	8.63	33.06	6.96	41.57	13.39	38.11	9.37
S–N (mm)	67.61	4.81	68.14	3.52	70.5	4.31	71.57	3.67	74.61	4.49	75.14	3.97
Max:MandOccP	0.64	0.45	0.72	0.32	0.38	0.48	0.52	0.28	0.28	0.29	0.35	0.23
Go-Me (mm)	63.57	4.69	62.94	7.34	72.71	8.48	70.22	6.57	80.86	6.52	76.06	5.32
MaxP/MandP (°)	27	3.37	27.5	5.09	24.29	5.06	24.44	4.95	18.86	4.1	23.17	4.82
MaxP/SN (°)	9.43	1.9	7.33	2.28	8.29	3.04	7.72	3.21	7.86	2.12	8.11	2.61
Go-Me/SN (°)	35.86	2.79	34.94	4.21	32.43	3.95	32.94	6.8	24.29	4.5	30.72	5.38
Ar-Go (mm)	40.14	3.18	38.22	3.49	48.14	6.34	42.67	4.93	56.14	4.56	49.28	4.84
AB/MandP (°)	66.71	5.94	66.33	4.16	67.29	5.62	69.17	4.6	66.71	9.32	67.33	5.31
Cond-Pog/FH (°)	39.71	3.99	40.35	4.01	42.57	3.78	42.75	3.91	42.71	4.68	43.76	2.97
APDI (°)	90.43	7.7	85.53	3.41	88.57	7.21	85	4.43	94.86	6.67	89.53	4.12
Me-Go-N (°)	73.71	2.93	69.83	16.02	73.14	5.46	72.78	4.28	71.43	5.47	72.61	4.1
FH/S-Gn (°)	51.71	5.38	52.06	5.33	52.43	1.62	55.38	4.54	54.86	5.46	55.29	3.08
Cond-Pog (mm)	102.43	9.43	100.76	9.68	117.57	12.62	109.65	12.5	126.57	11.91	118.72	14.59
Cranial base angle (°)	124.57	4.39	119.56	4.89	123.57	1.81	120.89	4.71	124.29	5.99	120.39	4.16
AB/facial plane (°)	3.57	2.7	2.5	1.92	3.29	2.43	2.5	2.01	3	2.71	3.22	2.34
Ant:post cranial b	2.31	0.22	2.24	0.25	2.1	0.23	2.16	0.26	2.05	0.29	2.07	0.22
NS/Gn (°)	78.06	5.07	75.14	9.65	79.11	5.61	77.29	10.26	89.57	12.23	79.67	5.94
AB/OccP (°)	81	5.39	83.11	4.56	85.29	5.79	85.61	3.58	79.29	8.99	84.94	4.08
Spa-Me (mm)	58	2.83	58.11	5.18	64.57	6.8	62.28	5.8	67.86	6.79	69	6.32
Upper gonial (°) angle	59.43	2.94	55.89	4.47	55.71	4.39	52	3.69	52	2	50.44	3.65
Upper-incisor incl. (°)	101	8.25	101.17	7.2	108.57	7.81	106	6.82	108	13.54	106.5	8.84
Lower-incisor incl. (°)	92.14	7.9	88.28	6.8	93.43	4.58	90.61	6.98	100	18.27	90.78	6.01
S–N:Spp-Spa	1.42	0.07	1.37	0.22	1.48	0.12	1.35	0.13	1.38	0.15	1.37	0.12
Go-Me:Spp-Spa	1.71	0.49	1.39	0.5	1.46	0.15	1.37	0.12	1.43	0.12	1.4	0.13
Go-Me:S–N	1.32	0.09	1.34	0.26	0.99	0.14	1.02	0.11	1.05	0.13	1.02	0.05

Table 2 presents a statistical comparison with age-matched normal Class I individuals from the literature [7, 23]. Only those 11 parameters are listed for which statistically significant differences were obtained. These parameters were closer to normal values in the success group at all times (*T*0, *T*1, and *T*2). At *T*0, significant differences were found for Go-Me (here the values in the success group were even below normal), APDI, NS-Gn, and overjet. At *T*1, significant differences were found for maxillomandibular differential, ratio of maxillary to mandibular occlusal plane, Ar-Go, FH/SGn, and NSGn. Of the significant parameters emerging at *T*2, Ar-Go (ramus height) showed marked increases both at *T*1 and *T*2; the angles between the maxillary and mandibular plane and Go-Me/SN decreased after *T*0. Dentally, the failure group exhibited greater mandibular anterior protrusion and more pronounced negative overjet. A majority of patients in the success group showed a position of point A anterior to the facial plane at *T*1. In the failure group, point B remained anterior to the facial plane at all times.

**Tab. 2:** Parameters showing significant differences between the sucess and failure group (expressed as p-values) to age-matched normal Class I individuals **Tab. 2** Parameter mit statistisch signifanten Unterschieden zwischen der Erfolgs/Misserfolgsgruppe (dargestellt als p-Werte) im Vergleich zu altersgematchten Klasse-I-Patienten

	*T*0	*T*1	*T*2
MM differential		0.034	
Max:MandOccP		0.008	
Go-Me	0.054		
MaxP/MandP			0.035
Go-Me/SN			0.010
Ar-Go		0.018	0.005
APDI	0.026		0.022
FH/S-Gn		0.033	
NS-Gn	0.072	0.011	0.007
Lower-incisor incl.			0.017
Overjet	0.035		0.017

*T0* pretreatment, *T1* posttreatment, *T2* 25-year follow-up

Table 3 lists the subset of parameters that showed significantly different developments in the success versus the failure group from *T*0 to *T*1 or from *T*1 to *T*2. Four parameters met this criterion, and all significantly different developments fell exclusively within the second period (*T*1 to *T*2). These findings indicate that both an overly vertical and an overly horizontal growth of the mandible will adversely affect the prognosis of Class III malocclusion.

**Tab. 3:** Parameters undergoing significantly different developments in the success versus the failure group from *T*0 to *T*1 or from *T*1 to *T*2. **Tab. 3** Parameter mit signifikant unterschiedlichen Entwicklungen (dargestellt als p-Werte) in der Erfolgs/Misserfolgsgruppe von T0 nach T1 und von T1 nach T2

	*T*0–*T*1	*T*1–*T*2
MaxP/MandP	–	0.012
Go-Me/SN	–	0.011
AB/OccP	–	0.047
Overjet	–	0.032

*T0* pretreatment, *T1* posttreatment, *T2* 25-year follow-up

Results are expressed as *p* values

Table 4 lists the descriptive statistical results seen with the four cast-based parameters, including overbite, overjet, and mandibular and maxillary intermolar width. The cast-based transverse evaluations revealed crossbite situations in 44% of cases at *T*0. Even though these situations had been resolved by *T*1, they relapsed in 16% by *T*2. Overjet values were clearly more negative in the failure group at *T*1 and *T*2. The mean values for mandibular intermolar width were (albeit not significantly) higher in the failure group.

**Tab. 4:** Statistically significant pretreatment (*T*0), posttreatment (*T*1) and 25-year follow-up (*T*2) data obtained in the success versus failure group for the four cast-based parameters measured **Tab. 4** Statistisch signifikante Modellbefunde in der Erfolgs- und in der Misserfolgsgruppe zu den Zeitpunkten *T*0 (vor Therapie), *T*1 (nach Therapie) und *T*2 (25 Jahre nach Therapie)

	Pretreatment values (*T*0)				Posttreatment values (*T*1)				25-year follow-up (*T*2)			
	Failure		Success		Failure		Success		Failure		Success	
	Mean	SD	Mean	SD	Mean	SD	Mean	SD	Mean	SD	Mean	SD
IMW mand (mm)	39.00	2.65	39.46	3.60	44.33	2.52	42.63	2.13	45.00	3.16	43.14	4.61
IMW max (mm)	41.00	1.00	42.67	3.31	48.00	1.73	48.25	1.98	49.80	3.03	48.29	2.58
Overbite (mm)	−0.17	4.62	−0.17	1.20	1.43	1.51	2.06	1.11	1.29	1.80	1.67	1.24
Overjet (mm)	−1.83	3.54	−0.56	2.57	−0.71	1.80	2.33	0.69	−2.29	1.50	1.89	0.76
*t* test (mm)	*p* = 0.347				*p* = 0.913				*p* = 0.028			

*IMW* intermolar width

Table 5 shows how the various prognathism types were related to treatment success. True mandibular prognathism was associated with a total success rate of 88%, but the outcome of treatment was better among female patients. Maxillary retrognathism accounted for 13% of cases and was treated very successfully (100%) without a gender difference. A majority of patients in the sample (55%) had combined forms of true mandibular prognathism and maxillary retrognathism. Failure was clearly more prevalent in this group regardless of gender (failure rate: 44%). Still, these data should be interpreted with due consideration given to the limited number of cases of our sample.

**Tab. 5:** Types of prognathism and treatment success **Tab. 5** Formen der Prognathie und Therapieerfolg

All patients (100%)					
True mandibular prognathism (32%)		Maxillary retrognathism (13%)		Combined pro- and retrognathism (55%)	
Success	Failure	Success	Failure	Success	Failure
88%	12%	100%	0%	56%	44%

From the patient data collected in the failure group, we derived the Graz Prognostic Score for Class III treatment outcome according to B. Wendl (inspired by M. Palmer). The main criteria for poor prognosis include the following:

- Male (+positive genetics),
- 10 years old,
- APDI: >90° ± 2°,
- MM differential: >32 mm (contribution of maxilla and mandible),
- Ar-Go: >42 mm,
- FH/S-Gn: <52°,
- NS/Gn: >85°,
- Severe negative overjet, and
- Protruded lower incisors and/or tongue habit.

Scores are calculated based on the number of criteria for poor prognosis present:

- 0–1: relatively good prognosis
- 2: treatment may be attempted
- 3–4: treatment requires patient (or parent/legal guardian) information about the increased risk of failure

Additional potential risk factors include the following:

- Go-Me: >64 mm,
- Cond-Pog: >100 mm,
- Cond-Gn: >112 mm,
- GoMe:SppSpa: >1.7,
- Maxillary intermolar width: <37 mm,
- Upper gonial angle: >60°,
- Gonial angle: >133°, and
- SNA angle: <76.

## Discussion

Björk [4] discovered that condylar growth is responsible for length development of the mandible (by constituting its center of growth) and defines the growth direction and position of the chin. Our study confirms that individual growth patterns are key to the prognosis of malocclusion. Ghiz et al. [11] retrospectively analyzed cephalometric landmarks and parameters by Björk, Odegaard and Riolo as predictors for Class III treatment outcome. They identified four parameters to forecast success: condylar position relative to cranial base, ramus length, mandibular length, and gonial angle. Also, they noted poor outcome in patients with a protruded mandible, short ramus, pronounced mandibular length, and large gonial angle. Each additional millimeter in Cond-Pog or ramus length was found to reduce or, respectively, increase the likelihood of successful outcome by a factor of 0.87 or 1.17. This is consistent with our own data for mandibular length, but not for ramus length, which, when excessive, predicted unfavorable outcome in our study. An excessive gonial angle will adversely impact outcomes, but the focus should be on the upper gonial angle. Even less favorable results should be expected given an excessive horizontal forward growth of the mandible.

By contrast, none of the parameters of maxillary size and position seemed to be a good outcome predictor. In some studies, a more posterior position of the maxilla was found to be suitable for this [22, 23]. Our analysis showed that the maxilla could be well controlled by treatment. The fact that the failure group showed greater increases in maxillomandibular differential may be attributed to a more pronounced growth of the mandible, thus, reflecting a growth pattern also found in untreated Class III patients [2, 6, 28]. According to Ko et al. [19], the improvements achieved by chincup treatment often cannot be maintained in patients showing a pronounced anteroposterior discrepancy, incisor compensation, and open bite tendency. For this reason, the parameters to be determined for prognosis should include the angle between AB line and mandibular plane, APDI, Wits appraisal, articular angle, gonial angle, ANB angle, facial convexity, AB to facial plane, and L1 to A-Pog. In our study, APDI likewise emerged as a significant parameter. Schuster et al. [31] identified Wits appraisal, palatal-plane inclination, and lower-incisor inclination as main predictors for future orthognathic surgery. Lower-incisor inclination, although in the direction of proclination, also emerged as a significant parameter in our study.

Tahmina et al. [34] reported that upward-and-forward rotation of the mandible, in conjunction with anteriorly directed growth and displacement, was associated with treatment failures among growing Class III patients after the pubertal growth spurt. Significant parameters were gonial angle, N-A-Pog angle, and angle from ramus line to SN plane. Moon et al. [24] reported less favorable prognoses of Class III treatment in patients with a large gonial angle and a vertical growth pattern, although mandibular size and anteroposterior relationships were similar to the findings in hypodivergent patients. The angle from AB to the mandibular plane was the most significant variable. Our data, too, emphasize the importance of vertical parameters, and Yashida et al. [36] likewise showed that these were essential for the prognosis of chincup and maxillary protraction treatment of Class III patients. Zentner et al. [37] identified the size ratio between the upper and lower apical bases as the best predictor.

Baccetti et al. [1] indicated increased ramus height, acute craniobasal angle, and steep mandibular plane to be prognostically unfavorable. Ferro et al. [8] identified four significant parameters, namely Wits appraisal, overbite, SNA, and ANB. Overbite also emerged as a potential predictor from our study. Franchi et al. [9] reported significant differences for CondAx-SBL, mandibular to palatal plane, and mandibular intermolar width. In our study, the success and failure group showed a significantly different relationship of the mandibular relative to the palatal plane (this angle became smaller). Also, the mandibular intermolar widths were larger in the failure group, although not significantly so. Ghiz et al. [11] identified four potential predictors for successful outcome: position of condyle relative to craniobasal plane, length of ramus, length of mandible, and gonial angle. While our study confirms some of these findings, it is fair to conclude in accordance with Fudalej et al. [10] that a precise forecasting of treatment outcomes in Class III patients remains questionable. There is a need for evidence-based data from prospective studies.

## Conclusions

- When maxillary retrognathism was the main feature of class III malocclusion, this was associated with relatively good treatment success.
- Combined mandibular prognathism and maxillary retrognathism was associated with clearly more treatment failures regardless of gender. Treatment outcome was difficult to predict in these cases, although this was also dependant on the extent of the skeletal malposition present. It is suggested in these combined Class III situations that close attention should be paid to the diagnostic and prognostic parameters identified in the present study.
- True mandibular prognathism was associated with clearly better outcome among female patients. This finding should, for course, be interpreted with due caution given to the limited number of cases of our sample.
- Transverse width of the maxilla should be treated with overcorrection and, given our finding of a 16% relapse rate, should be followed by an extended retention period.
- The Graz Prognostic Score according to Brigitte Wendl developed from our failure group should be assessed in clinical practice and ideally be verified in prospective studies.
